# Crystal structure of imidazo[1,5-*a*]pyridinium-based hybrid salt (C_13_H_12_N_3_)_2_[MnCl_4_]

**DOI:** 10.1107/S2056989020001425

**Published:** 2020-02-06

**Authors:** Olga Yu. Vassilyeva, Elena A. Buvaylo, Vladimir N. Kokozay, Svitlana R. Petrusenko, Andrii K. Melnyk, Brian W. Skelton

**Affiliations:** a Department of Chemistry, Taras Shevchenko National University of Kyiv, 64/13 Volodymyrska Street, Kyiv 01601, Ukraine; b Institute for Sorption and Problems of Endoecology, the National Academy of Sciences of Ukraine, 13 General Naumov str., Kyiv 03164, Ukraine; c School of Molecular Sciences, M310, University of Western Australia, Perth, WA 6009, Australia

**Keywords:** crystal structure, Mn^II^, organic-inorganic hybrid, tetra­halometallate, π–π stacking

## Abstract

A new organic–inorganic hybrid salt [*L*]_2_[MnCl_4_] (I) where *L* ^+^ is the 2-methyl-3-(pyridin-2-yl)imidazo[1,5-*a*]pyridinium cation, is built of discrete organic cations and tetra­chlorido­manganate(II) anions. The *L* ^+^ cation was formed *in situ* in the oxidative cyclo­condensation of 2-pyridine­carbaldehyde and CH_3_NH_2_·HCl in methanol. The structure was refined as a two-component twin using *PLATON* (Spek, 2020 ▸) to de-twin the data. The twin law (−1 0 0 0 − 1 0 0.5 0 1) was applied in the refinement where the twin component fraction refined to 0.155 (1). The compound crystallizes in the space group *P*2_1_/*c* with two crystallographically non-equivalent cations in the asymmetric unit, which possess similar structural conformations. The fused pyridinium and imidazolium rings of the cations are virtually coplanar [dihedral angles are 0.89 (18) and 0.78 (17)°]; the pendant pyridyl rings are twisted by 36.83 (14) and 36.14 (13)° with respect to the planes of the remaining atoms of the cations. The tetra­hedral MnCl_4_^2–^ anion is slightly distorted with the Mn—Cl distances falling in the range 2.3469 (10)–2.3941 (9) Å. The distortion value of 0.044 relative to the ideal tetra­hedron was obtained by continuous shape measurement (CShM) analysis. In the crystal, the cations and anions form separate stacks propagating along the *a-*axis direction. The organic cations display weak π–π stacking. The anions, which are stacked identically one above the other, demonstrate loose packing; the minimum Mn⋯Mn separation in the cation stack is approximately 7.49 Å. The investigation of the fluorescent properties of a powdered sample of (I) showed no emission. X-band EPR data for (I) at 293 and 77 K revealed broad fine structure signals, indicating moderate zero-field splitting.

## Abstract 2

The replacement of Zn^II^ with Mn^II^ ions in the hybrid structure, which also changed the space group from ortho­rhom­bic *Pbca* with *Z* = 8 to monoclinic *P*2_1_/c with *Z* = 4, quenched the fluorescence emission of the hybrid material.

## Chemical context   

Salts comprised of organic cations (*A*) and halometallate anions are a highly promising class of compounds within the more general domain of organic–inorganic hybrid materials. Hybrid salts *A*_2_[*M*Hal_4_] based on tetra­hedral anions of divalent transition metal ions (*M* = Zn, Mn, Co, Fe, Cd) can exhibit thermochromism (Kelley *et al.*, 2015 ▸) and multiferroic properties (Kapustianyk *et al.*, 2015 ▸) as well as acting as mol­ecular switchable dielectrics (Ji *et al.*, 2018 ▸) and ionic liquids (Miao *et al.*, 2011 ▸). Monovalent organic cations, where size, shape and electronic structure can be varied over wide limits, are a valuable tool for introducing useful properties into the hybrid structure. Heterocycles with the imidazo[1,5-*a*]pyridine skeleton have been identified as highly emissive fluoro­phores that render them suitable for optoelectronic devices (Hutt *et al.*, 2012 ▸; Yagishita *et al.*, 2018 ▸). Incorporation of the imidazo[1,5-*a*]pyridinium moiety in the hybrid structure is expected to extend the applications of the organic material, and also address such issues as mechanical properties, chemical resistance, thermal stability, *etc*., that limit the applicability of pure organics.
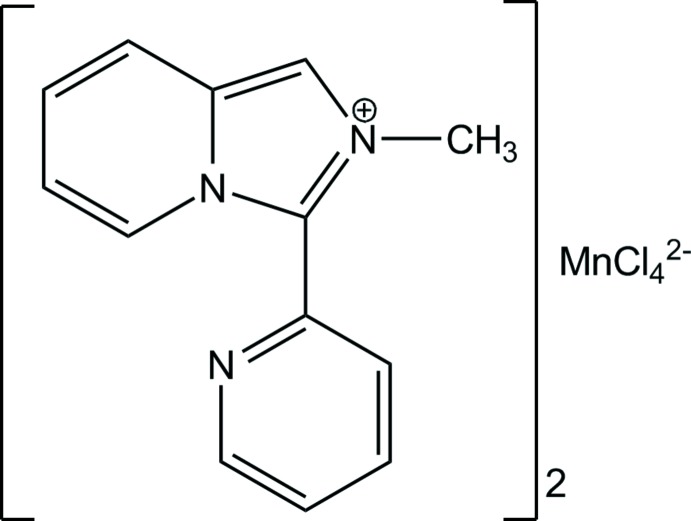


We have previously shown that the introduction of tetra­chloro­zincate anions significantly changes the optical behaviour of [*L*][Cl]·1.5H_2_O, where *L* ^+^ is the 2-methyl-3-(pyridin-2-yl)imidazo[1,5-*a*]pyridinium cation, in the solid state, also improving the thermal stability of the resulting hybrid salt (Buvaylo *et al.*, 2015 ▸). The *L* ^+^ cation was formed *in situ* in the oxidative cyclo­condensation of 2-pyridine­carbaldehyde (2-PCA) and CH_3_NH_2_·HCl in methanol. Upon excitation at 370 nm, a strong fluorescence was observed for [*L*][Cl]·1.5H_2_O at 406 nm, while [*L*]_2_[ZnCl_4_] [CSD (Groom *et al.*, 2016 ▸) refcode HUMHII; Buvaylo *et al.*, 2015 ▸] showed an intense blue-light fluorescence peak at 455 nm. Recent findings by Wei and coworkers on the unusual photoluminescence behaviour of tetra­hedral manganese(II) hybrid compounds, (*N*-methyl­piperidinium)MnCl_4_ and (*N*-methyl­pyrrol­id­in­ium)MnCl_4_ (Wei *et al.*, 2018 ▸), prompted us to synthesize the new organic–inorganic hybrid salt [*L*]_2_[MnCl_4_] (I) by the reaction of 2-PCA, CH_3_NH_2_·HCl and MnCl_2_·4H_2_O in CH_3_OH.

The investigation of the fluorescent properties of a powdered sample of (I) at room temperature under experimental conditions similar to those for [*L*]_2_[ZnCl_4_] showed no emission. The replacement of Zn^II^ with Mn^II^ ions in the hybrid structure, which also changed the space group from ortho­rhom­bic *Pbca* with Z = 8 to monoclinic *P*2_1_/c with Z = 4, quenched the fluorescence emission.

## Structural commentary   

**Figure 1:**
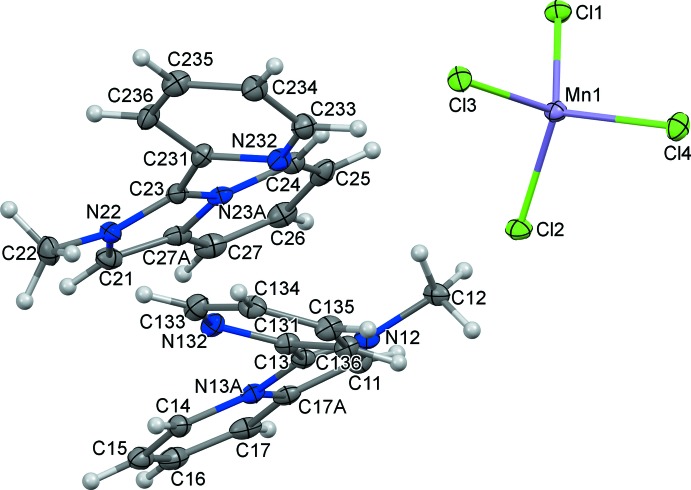
The mol­ecular structure and principal labelling of [*L*]_2_[MnCl_4_] (I) with displacement ellipsoids drawn at the 50% probability level.

Monoclinic crystals of [*L*]_2_[MnCl_4_] are built of discrete organic cations and tetra­chlorido­manganate(II) anions (Fig. 1 ▸). In the asymmetric unit, there are two crystallographically non-equivalent cations, *L*1 (N12, N13*A*) and *L*2 (N22, N23*A*), with similar structural conformations. The six-membered rings in the flattened imidazo[1,5-*a*]pyridinium cores have the expected bond lengths; the N/C—C bond distances in the imidazolium entities fall in the range 1.352 (4)–1.400 (4) Å. Both nitro­gen atoms in *L*1 and *L*2 are planar, showing the sum of the three angles to be 360°. The fused pyridinium and imidazolium rings of the cations are virtually coplanar with dihedral angles of 0.89 (18) (*L*1) and 0.78 (17)° (*L*2). The pendant pyridyl rings are twisted by 36.83 (14) and 36.14 (13)° with respect to the planes of the remaining atoms of the cations for *L*1 and *L*2, respectively. The geometric parameters of the cations closely resemble those found in the related organic–inorganic hybrids [*L*]_2_[*M*Cl_4_], where *M* = Co^II^, Zn^II^, and [*L*]_2_[CdI_4_] (Buvaylo *et al.*, 2015 ▸; Vassilyeva *et al.*, 2019*a* ▸,*b* ▸).

The tetra­hedral MnCl_4_^2–^ ion is slightly distorted. The Mn—Cl distances fall in the range 2.3469 (10)–2.3941 (9) Å and the Cl—Mn—Cl angles vary from 107.60 (3) to 112.95 (4)° (Table 1 ▸). The maximum differences in the lengths and angles are 0.047 Å and 5.35°, respectively. The distortion value of 0.044 relative to the ideal tetra­hedron obtained by the continuous shape measurement (CShM) analysis using the *SHAPE 2.1* program (Casanova *et al.*, 2005 ▸) supports a low degree of deformation.

## Supra­molecular features   

**Figure 2:**
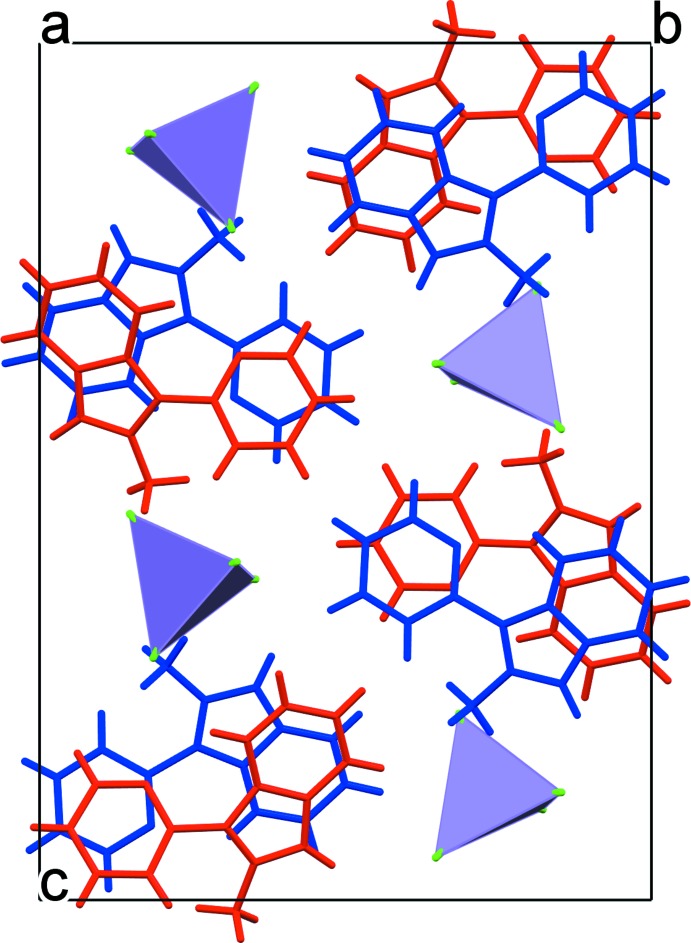
A fragment of the crystal packing of [*L*]_2_[MnCl_4_] (I) viewed along the *a* axis. The *L*1 and *L*2 cations are shown in blue and red, respectively.

In the crystal, the cations and anions form separate stacks propagating along the *a-*axis direction (Fig. 2 ▸). The alternating *L*1 and *L*2 organic cations display offset π–π stacking between the six-membered rings of the fused cores with the ring-centroid distances of 3.556 (2) and 4.0410 (2) Å. The aromatic stacking between the neighbouring pendant pyridyl rings of *L*1 and *L*2, which are twisted with respect to each other by 19.41 (17)° is also weak [the ring-centroid separations are 3.724 (2) and 3.956 (2) Å]. The anions, which are stacked identically one above the other, demonstrate loose packing: the shortest distance between the Cl atoms of adjacent anions of 3.79 Å is larger than double the chloride ionic radius (3.62 Å; Shannon, 1976 ▸). As a consequence, the minimum Mn⋯Mn separation in the cation stack is approximately 7.49 Å.

Classical hydrogen-bonding inter­actions are absent in (I). There are five C—H⋯Cl contacts between the cations and adjacent MnCl_4_^2–^ anions shorter than the van der Waals contact limit of 2.95 Å (Table 2 ▸). The closest cation–anion distance (C24—H24⋯Cl3—Mn) is 2.64 Å.

## Database survey   

A survey of the Cambridge Structural Database (CSD, Version 5.40, Oct 2019; Groom *et al.*, 2016 ▸) reveals that crystal structures containing an *L* ^+^ cation comprise the structures of two ligands in a salt form, three tetrahalo­metallates [*L*]_2_[*M*Cl_4_] and [*L*]_2_[CdI_4_], as well as two mol­ecular complexes [*ML*Cl_3_] (*M* = Co^II^ and Zn^II^) published by our group. While the latter are isostructural, the four hybrid tetra­halo­metallates including (I) possess different unit-cell parameters. The organic–inorganic hybrids exhibit either pseudo-layered structures with alternating layers of organic cations and of tetrahalo­metallate anions or are built of cations and anions arranged in separate stacks.

The imidazo[1,5-*a*]pyridinium core can be modified with various substituents on the aromatic rings and N(CH_3_) atom. Crystal structures of ten organic salts with *L* ^+^ derivatives as cations but no organic–inorganic hybrids or metal complexes are known. UREYIA (Türkyılmaz *et al.*, 2011 ▸) and YIHFEB (Mitra *et al.*, 2007 ▸), which bear ethyl­imidazolium and chloro­phenyl substituents, respectively, instead of the methyl group in *L* ^+^ are the most closely related. The lack of the methyl group turns *L* ^+^ into a neutral mol­ecule *L*′ that acts as a *κ* ^2^(*N*,*N*) chelate ligand, forming the mol­ecular Mn^II^ complex [Mn{S_2_P(OEt)_2_}_2_(*L*′)] (Álvarez *et al.*, 2012 ▸).

## IR and EPR spectroscopy measurements   

The IR spectrum of (I) is very similar to those of [*L*]_2_[CoCl_4_] and [*L*]_2_[ZnCl_4_] (Vassilyeva *et al.*, 2019*a* ▸; Buvaylo *et al.*, 2015 ▸) and shows a distinctive pattern that can be considered characteristic of *L* ^+^ (see supporting information). It includes intense absorption in the aromatic =C—H stretching region (3136–3012 cm^−1^) with several narrow peaks, weak bands below 3000 cm^−1^ due to alkyl –C—H stretching, sharp bands of medium intensity at 1650, 1586, 1516, 1470 and 1422 cm^−1^ associated with heterocyclic ring stretching, a very strong band at 780 cm^−1^ and two less strong absorptions in the out-of-plane C—H bending region 800–600 cm^−1^ (peaks at 742 and 664 cm^−1^). The remarkable feature of the spectrum is a gap in absorbance from 1650 to 1586 cm^−1^.

**Figure 3:**
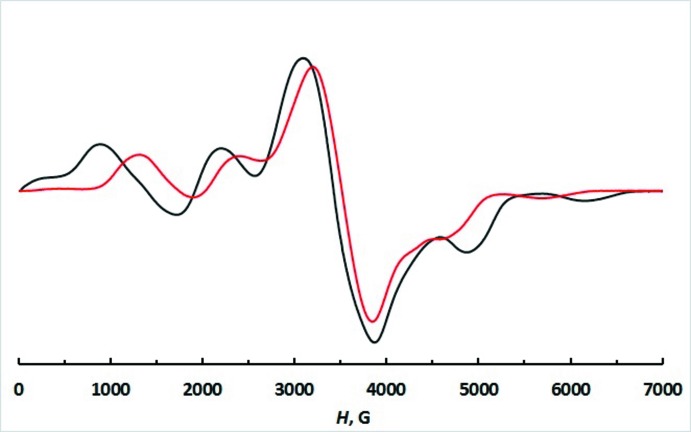
X-band EPR spectra of [*L*]_2_[MnCl_4_] (I) in the solid state at 293 (red) and 77 K (black).

The electronic structure of (I) was probed through X-band EPR spectroscopy at room temperature (r.t.) and 77 K. The EPR spectra of the neat powder sample are temperature-dependent (Fig. 3 ▸). At both temperatures, they are dominated by a strong line at 3500 G (*g*_eff_ ∼2) flanked by broad fine structure signals at approximately 900, 2200, 5000 and 6000 G (77 K; *g*_eff_ ∼7.97, 3.20, 1.42 and 1.16, respectively). The outer lines indicate zero-field splitting (ZFS) of the spin states for the high-spin *d* ^5^ metal ion. As expected for neat-powder EPR spectra, the ^55^Mn hyperfine structure due to the coupling of the unpaired electron spins with the *I* = 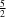 ^55^Mn nucleus is not resolved. The inter­molecular dipole–dipole inter­actions and the *D-*strain (*D* is the axial ZFS parameter) broaden the lines, thus preventing observation of the hyperfine structure in the spectra of neat powders (Duboc *et al.*, 2010 ▸). Computer simulation of the 77 K spectrum performed with the program *SPIN* (*S* > 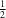; Ozarowski, 2019 ▸) yielded axial and rhombic ZFS parameters *D* of 0.062 cm^−1^ and *E* close to *D*/3, respectively. The highest field lines at ∼6000 and 5000 G result from a mixture of the *Z* and *Y* transitions |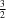 > → |
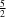
> and |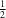 > → |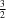 >, respectively.

For high-spin Mn^II^ complexes, a very small anisotropy of the Zeeman inter­action leads to *g* values close to 2, and the shape of the spectra depends on the ZFS terms only (Pilbrow, 1990 ▸). ZFS is highly sensitive to the coordination environment of the metal ion and if all bonds in the MnCl_4_ tetra­hedron are equal, one may expect only a strong and broadened single resonance line at *g* = 2 recorded in the EPR spectra. Indeed, one isotropic line with an unchanged linewidth of about 100 mT and a *g*-value of 2.0039 was observed in the X-band EPR spectrum of the organic–inorganic hybrid [(CH_3_)_4_N]_2_MnCl_4_ from 400 down to 20 K (Köksal *et al.*, 1999 ▸). The EPR spectra of another hybrid, [(C_2_H_5_)_4_N]_2_MnCl_4_, also consist of a broadened line with the isotropic *g*-value of 2.001 (3) in the temperature range 170–300 K (Ostrowski & Ciżman, 2008 ▸). The appearance of fine structure in the spectrum of (I) needs further study that requires the high-field/high-frequency EPR spectroscopy experiments to be undertaken at lower temperatures (Gagnon *et al.*, 2019 ▸).

## Synthesis and crystallization   

2-PCA (0.38 ml, 4 mmol) was stirred with CH_3_NH_2_·HCl (0.27 g, 4 mmol) in 20 ml of methanol in a 50 ml conical flask at room temperature for half an hour. The resultant yellow solution was left in open air overnight and used as the ligand without further purification. Dry MnCl_2_·4H_2_O (0.40 g, 2 mmol) was added to the solution of the ligand (which had turned olive) and the mixture was magnetically stirred under mild heating for 20 min to ensure dissolution of the metal salt. The resulting solution was filtered and left to cool at r.t. Colourless needles of (I) suitable for X-ray analysis were deposited next day. They were filtered off, washed with diethyl ether and finally dried in air. More product was obtained upon slow evaporation in air of the mother liquor. Total yield: 88%. Analysis calculated for (I), C_26_H_24_Cl_4_N_6_Mn (617.25): C, 50.59; H 3.92; N 13.62%. Found: C 50.69; H 3.75; N 13.39%. IR (ν, cm^−1^, KBr): 3402 (*br*), 3168, 3136, 3110, 3090, 3054, 3012, 2952, 2922, 1650, 1586, 1568, 1528 (*sh*), 1516, 1470, 1422, 1366, 1332, 1252, 1190, 1156, 1104, 1040, 992, 942, 780 (*vs*), 742 (*s*), 664, 610, 556, 500, 434.

## Refinement   

Crystal data, data collection and structure refinement details are summarized in Table 3 ▸. The structure was refined as a two-component twin using *PLATON* (Spek, 2020 ▸) to de-twin the data. The twin law (−1 0 0 0 − 1 0 0.5 0 1) was applied in the refinement where the twin component fraction refined to 0.155 (1). Anisotropic displacement parameters were employed for the non-hydrogen atoms. All hydrogen atoms were added at calculated positions and refined by use of a riding model with isotropic displacement parameters based on those of the parent atom (C—H = 0.95 Å, *U*_iso_(H) = 1.2*U*_eq_C for CH, C—H = 0.98 Å, *U*_iso_(H) = 1.5*U*_eq_C for CH_3_).

## Supplementary Material

Crystal structure: contains datablock(s) I, global. DOI: 10.1107/S2056989020001425/lh5945sup1.cif

Structure factors: contains datablock(s) I. DOI: 10.1107/S2056989020001425/lh5945Isup2.hkl

IR spectrum. DOI: 10.1107/S2056989020001425/lh5945sup3.pdf

CCDC reference: 1955880

Additional supporting information:  crystallographic information; 3D view; checkCIF report

## Figures and Tables

**Table 1:** Selected geometric parameters (Å, °)

Mn1—Cl1	2.3469 (10)	Mn1—Cl3	2.3779 (9)
Mn1—Cl2	2.3585 (9)	Mn1—Cl4	2.3941 (9)
			
Cl1—Mn1—Cl2	108.62 (3)	Cl1—Mn1—Cl4	112.95 (4)
Cl1—Mn1—Cl3	108.44 (3)	Cl2—Mn1—Cl4	110.60 (3)
Cl2—Mn1—Cl3	108.51 (3)	Cl3—Mn1—Cl4	107.60 (3)

**Table 2:** Hydrogen-bond geometry (Å, °)

*D*—H⋯*A*	*D*—H	H⋯*A*	*D*⋯*A*	*D*—H⋯*A*
C12—H12*B*⋯Cl2	0.98	2.79	3.746 (4)	167
C17—H17⋯Cl3^i^	0.95	2.68	3.450 (3)	139
C21—H21⋯Cl4^i^	0.95	2.82	3.625 (3)	143
C22—H22*A*⋯Cl1^ii^	0.98	2.79	3.378 (4)	120
C24—H24⋯Cl3	0.95	2.64	3.450 (4)	143

Symmetry codes: (i) 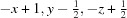; (ii) 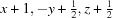.

**Table 3:** Experimental details

Crystal data	
Chemical formula	(C_13_H_12_N_3_)_2_[MnCl_4_]
*M*_r_	617.25
Crystal system, space group	Monoclinic, *P*2_1_/*c*
Temperature (K)	100
*a*, *b*, *c* (Å)	7.4892 (1), 15.9488 (4), 22.4266 (5)
β (°)	94.896 (2)
*V* (Å^3^)	2668.94 (10)
*Z*	4
Radiation type	Mo *K*α
μ (mm^−1^)	0.92
Crystal size (mm)	0.36 × 0.1 × 0.08

Data collection	
Diffractometer	Oxford Diffraction Gemini diffractometer
Absorption correction	Analytical (*CrysAlis PRO*; Rigaku OD, 2016 ▸)
*T*_min_, *T*_max_	0.753, 0.942
No. of measured, independent and observed [*I* > 2σ(*I*)] reflections	7856, 7856, 6781
*R*_int_	0.069
(sin θ/λ)_max_ (Å^−1^)	0.705

Refinement	
*R*[*F* ^2^ > 2σ(*F* ^2^)], *wR*(*F* ^2^), *S*	0.057, 0.186, 1.15
No. of reflections	7856
No. of parameters	337
H-atom treatment	H-atom parameters constrained
Δρ_max_, Δρ_min_ (e Å^−3^)	0.58, −0.69

Computer programs: *CrysAlis PRO* (Rigaku OD, 2016 ▸), *SIR92* (Altomare *et al.*, 1994 ▸), *SHELXL2017* (Sheldrick, 2015 ▸), *Mercury* (Macrae *et al.*, 2006 ▸) and *WinGX* (Farrugia, 2012 ▸).

## supplementary crystallographic information

### Crystal data

**Table:**

(C_13_H_12_N_3_)_2_[MnCl_4_]	*F*(000) = 1260
*M**_r_* = 617.25	*D*_x_ = 1.536 Mg m^−^^3^
Monoclinic, *P*2_1_/*c*	Mo *K*α radiation, λ = 0.71073 Å
Hall symbol: -P 2ybc	Cell parameters from 10548 reflections
*a* = 7.4892 (1) Å	θ = 3.6–34.6°
*b* = 15.9488 (4) Å	µ = 0.92 mm^−^^1^
*c* = 22.4266 (5) Å	*T* = 100 K
β = 94.896 (2)°	Needle, colourless
*V* = 2668.94 (10) Å^3^	0.36 × 0.1 × 0.08 mm
*Z* = 4	

### Data collection

**Table:**

Oxford Diffraction Gemini diffractometer	7856 independent reflections
Graphite monochromator	6781 reflections with *I* > 2σ(*I*)
Detector resolution: 10.4738 pixels mm^-1^	*R*_int_ = 0.069
ω scans	θ_max_ = 30.1°, θ_min_ = 3.6°
Absorption correction: analytical (CrysAlis PRO; Rigaku OD, 2016)	*h* = −10→10
*T*_min_ = 0.753, *T*_max_ = 0.942	*k* = −22→22
7856 measured reflections	*l* = −4→31

### Refinement

**Table:**

Refinement on *F*^2^	0 restraints
Least-squares matrix: full	Hydrogen site location: inferred from neighbouring sites
*R*[*F*^2^ > 2σ(*F*^2^)] = 0.057	H-atom parameters constrained
*wR*(*F*^2^) = 0.186	*w* = 1/[σ^2^(*F*_o_^2^) + (0.106*P*)^2^ + 2.4383*P*] where *P* = (*F*_o_^2^ + 2*F*_c_^2^)/3
*S* = 1.15	(Δ/σ)_max_ < 0.001
7856 reflections	Δρ_max_ = 0.58 e Å^−^^3^
337 parameters	Δρ_min_ = −0.69 e Å^−^^3^

### Special details

**Table:**

Geometry. All esds (except the esd in the dihedral angle between two l.s. planes) are estimated using the full covariance matrix. The cell esds are taken into account individually in the estimation of esds in distances, angles and torsion angles; correlations between esds in cell parameters are only used when they are defined by crystal symmetry. An approximate (isotropic) treatment of cell esds is used for estimating esds involving l.s. planes.
Refinement. Refined as a 2-component twin using PLATON to de-twin the data.

### Fractional atomic coordinates and isotropic or equivalent isotropic displacement parameters (Å^2^)

**Table:**

	*x*	*y*	*z*	*U*_iso_*/*U*_eq_	
Mn1	0.18155 (7)	0.24718 (3)	0.12270 (2)	0.01416 (13)	
Cl1	−0.09406 (11)	0.17752 (5)	0.10546 (4)	0.01936 (17)	
Cl2	0.41361 (11)	0.14691 (5)	0.12578 (3)	0.01805 (17)	
Cl3	0.18969 (12)	0.31455 (5)	0.21748 (3)	0.01903 (17)	
Cl4	0.22558 (12)	0.35257 (5)	0.04936 (4)	0.02042 (18)	
C11	0.7917 (5)	0.1463 (2)	0.25204 (15)	0.0163 (6)	
H11	0.743093	0.121304	0.215771	0.02*	
N12	0.7856 (4)	0.22928 (17)	0.26666 (12)	0.0149 (5)	
C12	0.6912 (5)	0.2909 (2)	0.22796 (16)	0.0207 (7)	
H12A	0.619181	0.327043	0.251868	0.031*	
H12B	0.612553	0.262038	0.197364	0.031*	
H12C	0.778253	0.32509	0.208559	0.031*	
C13	0.8700 (4)	0.2431 (2)	0.32148 (14)	0.0143 (6)	
N13A	0.9302 (4)	0.16698 (17)	0.34265 (12)	0.0139 (5)	
C14	1.0262 (5)	0.1464 (2)	0.39668 (15)	0.0171 (6)	
H14	1.05983	0.188652	0.425342	0.02*	
C15	1.0708 (5)	0.0651 (2)	0.40762 (15)	0.0197 (6)	
H15	1.137344	0.050607	0.444133	0.024*	
C16	1.0194 (5)	0.0007 (2)	0.36519 (16)	0.0211 (7)	
H16	1.04999	−0.056041	0.374005	0.025*	
C17	0.9270 (5)	0.0205 (2)	0.31227 (16)	0.0191 (6)	
H17	0.893137	−0.022108	0.283952	0.023*	
C17A	0.8815 (4)	0.1056 (2)	0.29965 (14)	0.0150 (6)	
C21	0.6189 (4)	0.0675 (2)	0.43736 (16)	0.0182 (6)	
H21	0.676985	0.022658	0.459085	0.022*	
N22	0.6065 (4)	0.14828 (18)	0.45664 (12)	0.0161 (5)	
C22	0.6955 (5)	0.1792 (2)	0.51343 (15)	0.0216 (7)	
H22A	0.734725	0.237161	0.508343	0.032*	
H22B	0.79976	0.143911	0.525234	0.032*	
H22C	0.61138	0.17701	0.544551	0.032*	
C23	0.5132 (4)	0.1952 (2)	0.41441 (14)	0.0153 (6)	
N23A	0.4677 (4)	0.14328 (17)	0.36724 (12)	0.0148 (5)	
C24	0.3675 (4)	0.1610 (2)	0.31331 (14)	0.0170 (6)	
H24	0.320143	0.215412	0.305083	0.02*	
C25	0.3403 (5)	0.0979 (2)	0.27312 (16)	0.0205 (7)	
H25	0.274347	0.108817	0.23585	0.025*	
C26	0.4079 (5)	0.0154 (2)	0.28524 (16)	0.0210 (7)	
H26	0.38685	−0.0273	0.255977	0.025*	
C27	0.5020 (5)	−0.0026 (2)	0.33805 (16)	0.0199 (6)	
H27	0.546507	−0.05758	0.34619	0.024*	
C27A	0.5324 (4)	0.0626 (2)	0.38099 (15)	0.0155 (6)	
C131	0.8898 (4)	0.3228 (2)	0.35385 (14)	0.0147 (6)	
C136	0.9174 (5)	0.3981 (2)	0.32441 (14)	0.0164 (6)	
H136	0.930694	0.398767	0.28266	0.02*	
C135	0.9251 (5)	0.4717 (2)	0.35712 (16)	0.0201 (7)	
H135	0.941632	0.524	0.338116	0.024*	
C134	0.9082 (5)	0.4677 (2)	0.41862 (16)	0.0200 (7)	
H134	0.911474	0.51718	0.442251	0.024*	
C133	0.8864 (5)	0.3896 (2)	0.44431 (14)	0.0187 (6)	
H133	0.87798	0.386788	0.486295	0.022*	
N132	0.8764 (4)	0.31762 (18)	0.41298 (13)	0.0180 (5)	
C231	0.4668 (4)	0.2846 (2)	0.41729 (14)	0.0143 (6)	
C236	0.4232 (5)	0.3215 (2)	0.47027 (15)	0.0185 (6)	
H236	0.421074	0.289572	0.505961	0.022*	
C235	0.3827 (5)	0.4069 (2)	0.46946 (16)	0.0219 (7)	
H235	0.35516	0.434543	0.505041	0.026*	
C234	0.3830 (5)	0.4504 (2)	0.41675 (17)	0.0234 (7)	
H234	0.355126	0.508481	0.415031	0.028*	
C233	0.4251 (5)	0.4074 (2)	0.36591 (17)	0.0219 (7)	
H233	0.423424	0.437845	0.329457	0.026*	
N232	0.4674 (4)	0.32664 (18)	0.36493 (13)	0.0173 (5)	

### Atomic displacement parameters (Å^2^)

**Table:**

	*U*^11^	*U*^22^	*U*^33^	*U*^12^	*U*^13^	*U*^23^
Mn1	0.0153 (2)	0.0128 (2)	0.0146 (2)	0.00108 (18)	0.00254 (17)	0.00018 (17)
Cl1	0.0168 (4)	0.0201 (4)	0.0213 (4)	−0.0013 (3)	0.0021 (3)	0.0024 (3)
Cl2	0.0175 (3)	0.0190 (4)	0.0177 (3)	0.0041 (3)	0.0019 (3)	−0.0019 (3)
Cl3	0.0244 (4)	0.0162 (3)	0.0166 (3)	0.0054 (3)	0.0023 (3)	−0.0013 (3)
Cl4	0.0271 (4)	0.0148 (3)	0.0200 (4)	0.0001 (3)	0.0054 (3)	0.0025 (3)
C11	0.0196 (15)	0.0131 (13)	0.0165 (14)	−0.0046 (12)	0.0028 (12)	−0.0042 (11)
N12	0.0161 (13)	0.0110 (11)	0.0176 (12)	−0.0003 (10)	0.0018 (10)	−0.0003 (9)
C12	0.0257 (17)	0.0140 (14)	0.0216 (15)	0.0030 (13)	−0.0032 (13)	0.0016 (12)
C13	0.0131 (13)	0.0138 (14)	0.0163 (14)	−0.0017 (11)	0.0044 (11)	−0.0008 (11)
N13A	0.0145 (12)	0.0124 (11)	0.0152 (12)	−0.0005 (10)	0.0041 (10)	−0.0004 (9)
C14	0.0158 (14)	0.0172 (15)	0.0181 (14)	0.0005 (12)	0.0004 (11)	0.0000 (12)
C15	0.0204 (16)	0.0178 (15)	0.0212 (15)	0.0031 (13)	0.0043 (12)	0.0043 (12)
C16	0.0199 (16)	0.0172 (15)	0.0277 (17)	0.0015 (13)	0.0104 (13)	0.0039 (13)
C17	0.0195 (16)	0.0125 (14)	0.0260 (16)	−0.0007 (12)	0.0068 (13)	−0.0016 (12)
C17A	0.0147 (14)	0.0135 (14)	0.0172 (14)	−0.0015 (11)	0.0035 (11)	−0.0030 (11)
C21	0.0143 (14)	0.0134 (14)	0.0266 (16)	0.0024 (12)	0.0006 (12)	0.0037 (12)
N22	0.0155 (12)	0.0166 (12)	0.0164 (12)	0.0005 (11)	0.0022 (10)	0.0013 (10)
C22	0.0224 (17)	0.0240 (17)	0.0174 (15)	−0.0004 (14)	−0.0037 (13)	0.0019 (13)
C23	0.0151 (14)	0.0169 (14)	0.0144 (13)	−0.0005 (12)	0.0044 (11)	−0.0005 (11)
N23A	0.0132 (12)	0.0130 (12)	0.0182 (12)	−0.0010 (10)	0.0011 (10)	−0.0003 (10)
C24	0.0158 (14)	0.0199 (15)	0.0155 (14)	−0.0026 (12)	0.0030 (11)	−0.0002 (12)
C25	0.0198 (16)	0.0217 (16)	0.0201 (15)	−0.0083 (14)	0.0026 (12)	−0.0015 (13)
C26	0.0217 (16)	0.0188 (15)	0.0235 (16)	−0.0056 (13)	0.0072 (13)	−0.0062 (13)
C27	0.0191 (16)	0.0128 (14)	0.0286 (17)	−0.0009 (12)	0.0057 (13)	−0.0008 (12)
C27A	0.0125 (13)	0.0133 (13)	0.0205 (15)	−0.0002 (11)	0.0007 (11)	−0.0003 (11)
C131	0.0129 (13)	0.0123 (13)	0.0187 (14)	−0.0019 (11)	0.0002 (11)	−0.0015 (11)
C136	0.0181 (15)	0.0146 (14)	0.0168 (14)	−0.0008 (12)	0.0029 (12)	−0.0005 (11)
C135	0.0208 (16)	0.0130 (14)	0.0265 (17)	0.0005 (13)	0.0018 (13)	0.0004 (12)
C134	0.0217 (16)	0.0117 (14)	0.0264 (17)	0.0010 (12)	0.0020 (13)	−0.0057 (12)
C133	0.0205 (16)	0.0227 (16)	0.0132 (13)	−0.0019 (13)	0.0025 (12)	−0.0026 (12)
N132	0.0182 (13)	0.0152 (13)	0.0212 (13)	−0.0017 (11)	0.0052 (11)	−0.0015 (10)
C231	0.0140 (14)	0.0127 (13)	0.0163 (14)	−0.0013 (11)	0.0016 (11)	−0.0002 (11)
C236	0.0203 (16)	0.0177 (15)	0.0186 (15)	−0.0012 (13)	0.0074 (12)	0.0013 (12)
C235	0.0255 (17)	0.0157 (15)	0.0258 (17)	−0.0015 (14)	0.0096 (14)	−0.0053 (13)
C234	0.0253 (17)	0.0121 (14)	0.0338 (19)	−0.0001 (13)	0.0085 (15)	−0.0013 (13)
C233	0.0226 (16)	0.0165 (15)	0.0266 (17)	0.0020 (13)	0.0024 (13)	0.0036 (13)
N232	0.0164 (13)	0.0148 (12)	0.0207 (13)	−0.0007 (11)	0.0015 (10)	0.0010 (10)

### Geometric parameters (Å, º)

**Table:**

Mn1—Cl1	2.3469 (10)	C23—N23A	1.364 (4)
Mn1—Cl2	2.3585 (9)	C23—C231	1.471 (5)
Mn1—Cl3	2.3779 (9)	N23A—C24	1.396 (4)
Mn1—Cl4	2.3941 (9)	N23A—C27A	1.400 (4)
C11—N12	1.366 (4)	C24—C25	1.355 (5)
C11—C17A	1.375 (5)	C24—H24	0.95
C11—H11	0.95	C25—C26	1.427 (5)
N12—C13	1.352 (4)	C25—H25	0.95
N12—C12	1.454 (4)	C26—C27	1.356 (5)
C12—H12A	0.98	C26—H26	0.95
C12—H12B	0.98	C27—C27A	1.423 (5)
C12—H12C	0.98	C27—H27	0.95
C13—N13A	1.367 (4)	C131—N132	1.341 (4)
C13—C131	1.465 (4)	C131—C136	1.394 (4)
N13A—C14	1.394 (4)	C136—C135	1.383 (5)
N13A—C17A	1.400 (4)	C136—H136	0.95
C14—C15	1.357 (5)	C135—C134	1.397 (5)
C14—H14	0.95	C135—H135	0.95
C15—C16	1.430 (5)	C134—C133	1.388 (5)
C15—H15	0.95	C134—H134	0.95
C16—C17	1.359 (5)	C133—N132	1.344 (4)
C16—H16	0.95	C133—H133	0.95
C17—C17A	1.422 (4)	C231—N232	1.353 (4)
C17—H17	0.95	C231—C236	1.390 (4)
C21—N22	1.364 (4)	C236—C235	1.394 (5)
C21—C27A	1.373 (5)	C236—H236	0.95
C21—H21	0.95	C235—C234	1.371 (5)
N22—C23	1.353 (4)	C235—H235	0.95
N22—C22	1.471 (4)	C234—C233	1.389 (5)
C22—H22A	0.98	C234—H234	0.95
C22—H22B	0.98	C233—N232	1.327 (4)
C22—H22C	0.98	C233—H233	0.95
			
Cl1—Mn1—Cl2	108.62 (3)	N22—C23—C231	128.0 (3)
Cl1—Mn1—Cl3	108.44 (3)	N23A—C23—C231	125.3 (3)
Cl2—Mn1—Cl3	108.51 (3)	C23—N23A—C24	129.0 (3)
Cl1—Mn1—Cl4	112.95 (4)	C23—N23A—C27A	109.2 (3)
Cl2—Mn1—Cl4	110.60 (3)	C24—N23A—C27A	121.8 (3)
Cl3—Mn1—Cl4	107.60 (3)	C25—C24—N23A	117.7 (3)
N12—C11—C17A	107.2 (3)	C25—C24—H24	121.1
N12—C11—H11	126.4	N23A—C24—H24	121.1
C17A—C11—H11	126.4	C24—C25—C26	121.8 (3)
C13—N12—C11	110.7 (3)	C24—C25—H25	119.1
C13—N12—C12	126.7 (3)	C26—C25—H25	119.1
C11—N12—C12	122.4 (3)	C27—C26—C25	120.8 (3)
N12—C12—H12A	109.5	C27—C26—H26	119.6
N12—C12—H12B	109.5	C25—C26—H26	119.6
H12A—C12—H12B	109.5	C26—C27—C27A	118.5 (3)
N12—C12—H12C	109.5	C26—C27—H27	120.8
H12A—C12—H12C	109.5	C27A—C27—H27	120.8
H12B—C12—H12C	109.5	C21—C27A—N23A	106.1 (3)
N12—C13—N13A	106.6 (3)	C21—C27A—C27	134.5 (3)
N12—C13—C131	127.8 (3)	N23A—C27A—C27	119.4 (3)
N13A—C13—C131	125.6 (3)	N132—C131—C136	123.3 (3)
C13—N13A—C14	129.9 (3)	N132—C131—C13	115.1 (3)
C13—N13A—C17A	108.9 (3)	C136—C131—C13	121.7 (3)
C14—N13A—C17A	121.2 (3)	C135—C136—C131	118.7 (3)
C15—C14—N13A	118.9 (3)	C135—C136—H136	120.6
C15—C14—H14	120.6	C131—C136—H136	120.6
N13A—C14—H14	120.6	C136—C135—C134	118.8 (3)
C14—C15—C16	121.2 (3)	C136—C135—H135	120.6
C14—C15—H15	119.4	C134—C135—H135	120.6
C16—C15—H15	119.4	C133—C134—C135	118.3 (3)
C17—C16—C15	120.1 (3)	C133—C134—H134	120.8
C17—C16—H16	119.9	C135—C134—H134	120.8
C15—C16—H16	119.9	N132—C133—C134	123.5 (3)
C16—C17—C17A	119.4 (3)	N132—C133—H133	118.2
C16—C17—H17	120.3	C134—C133—H133	118.2
C17A—C17—H17	120.3	C131—N132—C133	117.3 (3)
C11—C17A—N13A	106.6 (3)	N232—C231—C236	123.4 (3)
C11—C17A—C17	134.3 (3)	N232—C231—C23	115.1 (3)
N13A—C17A—C17	119.1 (3)	C236—C231—C23	121.5 (3)
N22—C21—C27A	107.8 (3)	C231—C236—C235	118.0 (3)
N22—C21—H21	126.1	C231—C236—H236	121
C27A—C21—H21	126.1	C235—C236—H236	121
C23—N22—C21	110.2 (3)	C234—C235—C236	119.3 (3)
C23—N22—C22	126.1 (3)	C234—C235—H235	120.4
C21—N22—C22	123.5 (3)	C236—C235—H235	120.4
N22—C22—H22A	109.5	C235—C234—C233	118.4 (3)
N22—C22—H22B	109.5	C235—C234—H234	120.8
H22A—C22—H22B	109.5	C233—C234—H234	120.8
N22—C22—H22C	109.5	N232—C233—C234	124.3 (3)
H22A—C22—H22C	109.5	N232—C233—H233	117.8
H22B—C22—H22C	109.5	C234—C233—H233	117.8
N22—C23—N23A	106.7 (3)	C233—N232—C231	116.6 (3)
			
C17A—C11—N12—C13	0.7 (4)	N23A—C24—C25—C26	1.1 (5)
C17A—C11—N12—C12	−176.4 (3)	C24—C25—C26—C27	0.3 (5)
C11—N12—C13—N13A	−0.5 (4)	C25—C26—C27—C27A	−0.3 (5)
C12—N12—C13—N13A	176.5 (3)	N22—C21—C27A—N23A	0.0 (4)
C11—N12—C13—C131	−178.6 (3)	N22—C21—C27A—C27	178.9 (4)
C12—N12—C13—C131	−1.6 (5)	C23—N23A—C27A—C21	−0.4 (4)
N12—C13—N13A—C14	179.6 (3)	C24—N23A—C27A—C21	−178.2 (3)
C131—C13—N13A—C14	−2.3 (5)	C23—N23A—C27A—C27	−179.6 (3)
N12—C13—N13A—C17A	0.1 (4)	C24—N23A—C27A—C27	2.6 (5)
C131—C13—N13A—C17A	178.2 (3)	C26—C27—C27A—C21	−180.0 (4)
C13—N13A—C14—C15	179.8 (3)	C26—C27—C27A—N23A	−1.1 (5)
C17A—N13A—C14—C15	−0.8 (5)	N12—C13—C131—N132	141.6 (3)
N13A—C14—C15—C16	−0.7 (5)	N13A—C13—C131—N132	−36.1 (5)
C14—C15—C16—C17	1.2 (5)	N12—C13—C131—C136	−37.3 (5)
C15—C16—C17—C17A	−0.2 (5)	N13A—C13—C131—C136	144.9 (3)
N12—C11—C17A—N13A	−0.7 (4)	N132—C131—C136—C135	−2.4 (5)
N12—C11—C17A—C17	178.3 (4)	C13—C131—C136—C135	176.5 (3)
C13—N13A—C17A—C11	0.4 (4)	C131—C136—C135—C134	1.1 (5)
C14—N13A—C17A—C11	−179.2 (3)	C136—C135—C134—C133	0.7 (5)
C13—N13A—C17A—C17	−178.8 (3)	C135—C134—C133—N132	−1.6 (5)
C14—N13A—C17A—C17	1.7 (5)	C136—C131—N132—C133	1.6 (5)
C16—C17—C17A—C11	180.0 (4)	C13—C131—N132—C133	−177.3 (3)
C16—C17—C17A—N13A	−1.2 (5)	C134—C133—N132—C131	0.4 (5)
C27A—C21—N22—C23	0.5 (4)	N22—C23—C231—N232	144.4 (3)
C27A—C21—N22—C22	−175.1 (3)	N23A—C23—C231—N232	−35.8 (5)
C21—N22—C23—N23A	−0.7 (4)	N22—C23—C231—C236	−36.6 (5)
C22—N22—C23—N23A	174.7 (3)	N23A—C23—C231—C236	143.2 (3)
C21—N22—C23—C231	179.0 (3)	N232—C231—C236—C235	−1.8 (5)
C22—N22—C23—C231	−5.5 (5)	C23—C231—C236—C235	179.3 (3)
N22—C23—N23A—C24	178.3 (3)	C231—C236—C235—C234	1.6 (5)
C231—C23—N23A—C24	−1.5 (5)	C236—C235—C234—C233	−0.4 (6)
N22—C23—N23A—C27A	0.7 (4)	C235—C234—C233—N232	−0.8 (6)
C231—C23—N23A—C27A	−179.1 (3)	C234—C233—N232—C231	0.7 (5)
C23—N23A—C24—C25	−179.9 (3)	C236—C231—N232—C233	0.6 (5)
C27A—N23A—C24—C25	−2.6 (5)	C23—C231—N232—C233	179.6 (3)

### Hydrogen-bond geometry (Å, º)

**Table:**

*D*—H···*A*	*D*—H	H···*A*	*D*···*A*	*D*—H···*A*
C12—H12*B*···Cl2	0.98	2.79	3.746 (4)	167
C14—H14···N132	0.95	2.48	2.986 (4)	114
C17—H17···Cl3^i^	0.95	2.68	3.450 (3)	139
C21—H21···Cl4^i^	0.95	2.82	3.625 (3)	143
C22—H22*A*···Cl1^ii^	0.98	2.79	3.378 (4)	120
C24—H24···Cl3	0.95	2.64	3.450 (4)	143
C24—H24···N232	0.95	2.43	2.955 (4)	115

Symmetry codes: (i) −*x*+1, *y*−1/2, −*z*+1/2; (ii) *x*+1, −*y*+1/2, *z*+1/2.
