# Supplementary figures and images for: Crystal structure of imidazo[1,5-a]pyridinium-based hybrid salt (C13H12N3)2[MnCl4]
Source: Acta Crystallogr E Crystallogr Commun. 2020 Feb 6;76(Pt 3):309–13. doi: 10.1107/S2056989020001425 (PMC7057380; doi:10.1107/S2056989020001425)

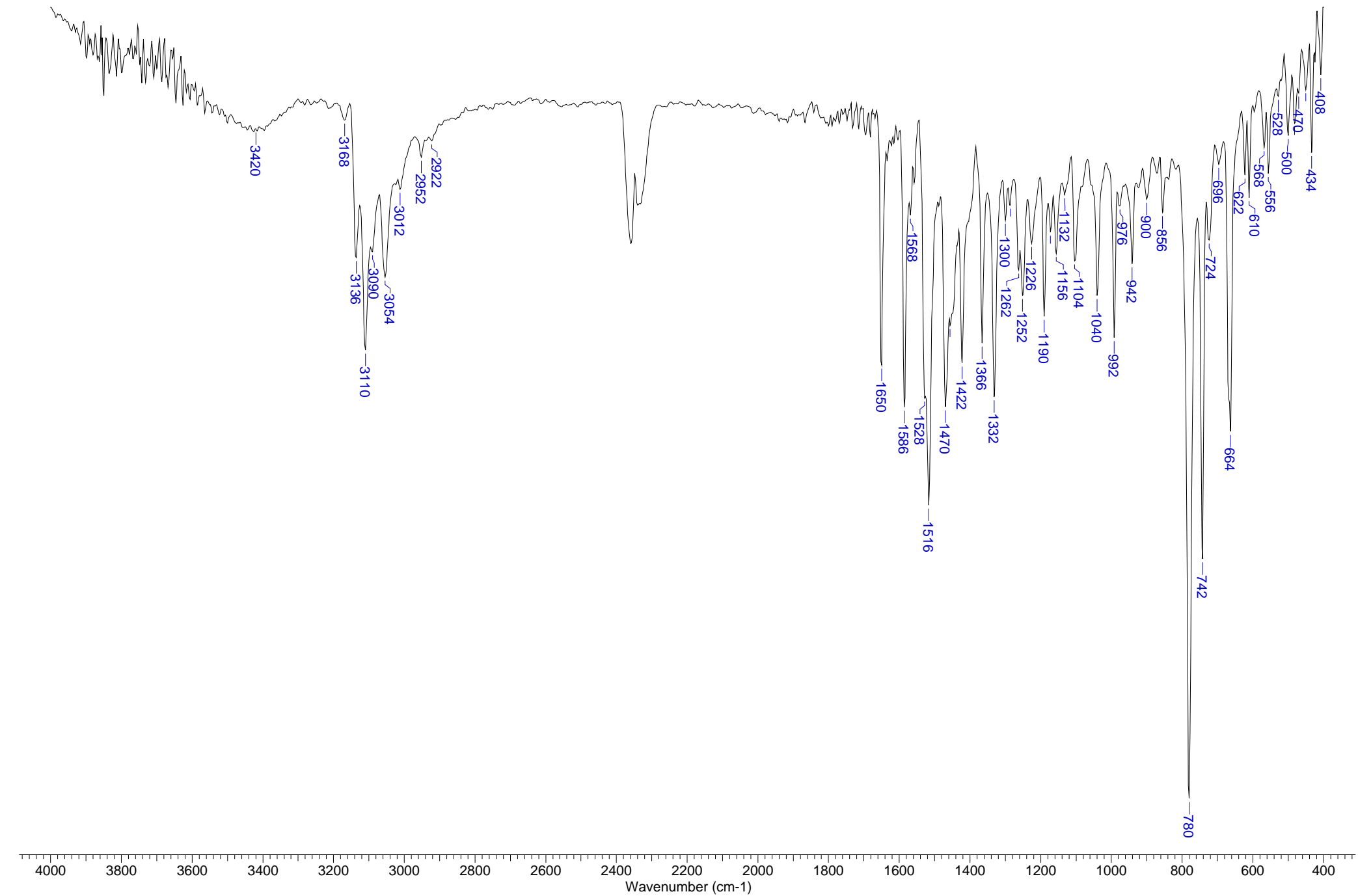

Supplement: Supplementary file 3 [file e-76-00309-sup3.pdf]
